# Active Middle Ear Implant Evoked Auditory Brainstem Response Intensity-Latency Characteristics

**DOI:** 10.3389/fneur.2021.739906

**Published:** 2022-01-20

**Authors:** Laura Fröhlich, Alexander Müller, Miriam H. Kropp, Parwis Mir-Salim, Oliver Dziemba, Tobias Oberhoffner, Stefan K. Plontke, Torsten Rahne

**Affiliations:** ^1^Department of Otorhinolaryngology, Head and Neck Surgery, Martin Luther University Halle-Wittenberg, Halle, Germany; ^2^Department of Otorhinolaryngology, Head and Neck Surgery, Friedrichshain Clinic, Vivantes Hearing Center, Berlin, Germany; ^3^Department of Otorhinolaryngology, Head and Neck Surgery, University Medicine of Greifswald, Greifswald, Germany; ^4^Department of Otorhinolaryngology, Head and Neck Surgery “Otto Körner”, Rostock University Medical Center, Rostock, Germany

**Keywords:** active middle ear implant, coupling efficiency, objective measures, auditory brainstem response, latency

## Abstract

**Objective:**

To analyze intensity-latency functions of intraoperative auditory evoked brainstem responses (ABRs) to stimulation by the Vibrant Soundbridge (VSB) active middle ear implant with respect to coupling efficiency, VSB evoked ABR thresholds, and coupling modality [oval window (OW) placement vs. Incus placement and vs. round window (RW) placement].

**Study Design:**

Exploratory study.

**Setting:**

Bi-centric study at tertiary referral centers.

**Patients:**

Twenty-four patients (10 female, 14 male, mean age: 58 years) who received a VSB.

**Outcome Measures:**

Wave-V intensity-latency functions of intraoperative VSB evoked ABRs using a modified audio processor programmed to preoperative bone conduction thresholds for stimulation. Threshold level correction to coupling efficiency and ABR thresholds. Individual plots and exponential function fits.

**Results:**

After ABR threshold level correction, the latency functions could be aligned. A large variance of latencies was observed at individual threshold level. Wave-V latency was longest in the Incus placement subgroup (9.73 ms, SD: 1.04) as compared to OW placement subgroup (9.47 ms, SD: 1.05), with the shortest latency in the RW placement subgroup (8.99 ms, SD: 0.68). For increasing stimulation levels, the variance decreased with intensity-latency function slopes converging toward a steady-state (saturation) latency caused by saturation of audio processor (stimulation) gain. Latency saturation was reached at a stimulation level of 50 dB nHL for the OW placement subgroup, 35 dB nHL for the Incus placement subgroup, and 30 dB nHL for the RW placement subgroup. The latency and saturation results indicated decreased dynamic range for RW placement, i.e., reverse stimulation.

**Conclusions:**

VSB evoked ABR wave-V intensity-latency function slopes were similar to acoustic stimulation at high stimulation levels with a shift toward longer latencies caused by audio processor signal delay. Saturation of latencies occurred for higher stimulation levels due to saturation of audio processor gain. Thus, the analysis of VSB evoked intensity-latency functions appears to allow for the objective assessment of a patient's individual dynamic range. This can further improve diagnostics as well as intraoperative and postoperative quality control.

## Introduction

Active middle ear implants (AMEI) are widely used for hearing rehabilitation in patients with sensorineural, conductive, and mixed hearing loss. This has become an appropriate solution for those who cannot be treated with conventional hearing aids due to technical issues such as feedback or sound distortion, or patient related issues like recurrent infections of the auditory canal (1). The Vibrant Soundbridge™ (VSB) (MED-EL, Innsbruck, Austria) is one of the available AMEI systems transforming sound into mechanical vibrations by its electromagnetic miniature floating mass transducer (FMT) (2, 3). The device was originally designed for treatment of sensorineural hearing loss where the only option for vibrational energy transfer was to couple the FMT to the long process of the incus (4). With the development of news couplers, the FMT can now be coupled to the long process (LP) of the incus (4), the short process (SP) and the head of the incus (5), the stapes suprastructure or the stapes footplate, i.e., the oval window (OW) (6, 7), and the round window (RW) membrane (8). The surgical procedure is referred to as vibroplasty. The performance of AMEIs is determined by biomechanical factors (9) as well as by the surgical procedure itself. With coupling location and coupling direction, which will be summarized and referred to as coupling modality here, the effective direction of stimulation varies. The output of AMEIs can be investigated by Laser-Doppler vibrometry which is also a feasible method to compare transfer functions for different coupling modalities (10). It has been shown that transfer functions for electromechanical stimulation by the FMT are different from acoustic stimulation and variations for different coupling modalities have been reported. Coupling to the stapes suprastructure or stapes footplate (OW placement) resulting in stimulation in the natural, normal direction (i.e., perpendicular to the OW) are biomechanically equally efficient (11). Regarding Incus placement, coupling to the incus body (SP) is a more complex coupling point, because in normal middle ears the malleus-incus motion rotation axis changes with frequency (12, 13). Thus, there is a risk of ineffective movements with FMT resulting in a reduction of the transfer function (9). However, this was not visible in experimental Laser-Doppler vibrometry measurements with the SP-Coupler (5). Similar velocity responses (transfer functions) have been obtained for coupling to the LP (14). In contrast, coupling to the RW (RW placement) results in reverse stimulation of the cochlea, which is a different mechanism compared to forward stimulation as was shown by intracochlear differential pressure measurements (15). Reverse stimulation was found to be less effective than stimulation at the stapes footplate (natural, normal direction) (16), and the efficiency of RW coupling was influenced significantly by technical and surgical factors (17).

Hearing improvement in patients treated with a VSB is highly dependent on sufficient energy transfer to the inner ear, i.e., on efficient coupling between the FMT and the middle ear structure or the RW (18). Clinically, the coupling efficiency can be quantified by the difference between vibroplasty *in situ* thresholds (vibrogram—VIB), which are measured as behavioral thresholds by ordinary pure-tone audiometry to stimulation via the implanted FMT, and bone conduction (BC) thresholds. Small differences indicate good coupling, i.e., efficient energy transmission to the inner ear without loss of energy. To determine the coupling efficiency objectively, auditory evoked potentials like auditory steady state responses (ASSRs) (19, 20), compound action potentials (CAPs) (21–23), and auditory brainstem responses (ABRs) (24–27) have been recorded in patients treated with a VSB. Custom-made experimental set-ups were used in all studies for providing stimulus transmission by the FMT. While most studies only aimed on relative measurements, it has recently been reported that coupling efficiency could also be determined quantitatively by measuring VSB evoked ABRs (26, 27). A modified AP404 audio processor (MED-EL, Innsbruck, Austria) programmed to preoperative BC thresholds and fitted with an insert earphone sound tube attached to the microphone aperture was used for providing stimulation by the implanted FMT. The VSB evoked ABR thresholds in this set-up have been shown to directly predict the mean coupling efficiency at 1, 2, and 4 kHz (3PTA_coupling_–pure tone average of coupling efficiency at 1, 2, and 4 kHz). However, VSB evoked ABR wave-V intensity-latency functions have not been evaluated so far with respect to coupling efficiency or other parameters.

ABR measurements, as first described by Jewett in 1970 (28), are regularly used today for threshold determination in infants and objective evaluation of the auditory pathway. ABR wave-V intensity-latency functions can be used to assess and differentiate between types and magnitude of hearing loss objectively (29–31). However, for clinical interpretations it has to be considered that intensity-latency functions are also dependent on EEG (electroencephalogram) filters, stimulation rate (32), the stimulus itself, and stimulation mode, i.e., longer latencies but comparable variance have been reported for stimulation by BC vs. air conduction (AC) (33). Typical stimuli for ABR recordings are clicks but also other stimuli such as specific chirps, e.g., the CE-Chirp®, have been developed in recent years (34). Studies have reported larger amplitudes for chirps as compared to traditional click stimuli helping in threshold determination (35, 36). Due to the variability of ABRs and ABR wave-V intensity-latency functions for different stimulation parameters, normative data were recorded for clicks and CE-Chirps (34, 37–39), respectively.

With the growing number of implantation of AMEIs and other implantable hearing devices such as cochlear implants, the field of auditory evoked potential recording becomes increasingly important in objective assessment of implant performance and outcome prediction. Thus, research in the field is urgently necessary. For cochlear implants, the use of auditory evoked potentials to electrical stimulation is already part of clinical routine measurements [see for example (40) for an overview]. Normative data for electrically evoked ABR wave V latencies and interpeak latencies have also been established (41). However, standardized ABR peak V latencies as well as intensity-latency functions obtained by aided stimulation with AMEIs are currently lacking in the literature. The studies investigating VSB evoked ABRs did not report latency data but focused on thresholds to predict coupling efficiency (25–27). In electrocochleography measurements, Colletti et al. found reduced CAP latencies and higher amplitudes in case of better coupling (23). Verhaert et al. investigated ABRs to stimulation by a Codacs™ implant (Cochlear, Sydney, Australia) in three subjects and reported latencies longer than for normal hearing subjects to acoustic stimulation with some interindividual variability (42). Wave-V intensity-latency functions were not analyzed in their study. Cebulla et al. described an optimized CE-Chirp for ABR measurements with the VSB and reported larger amplitudes compared to using standard CE-Chirps but did not elaborate on intensity-latency functions (24).

The objective of this study was to analyze and describe VSB evoked ABR wave-V intensity-latency functions with respect to coupling efficiency, response thresholds, and coupling modality (OW placement vs. Incus placement and vs. RW placement).

## Materials and Methods

### Study Design and Participants

An exploratory study was conducted on adult patients who were regularly scheduled for hearing rehabilitation with the VSB between September 2017 and March 2021. The audiological and patient criteria for implantation as provided by the manufacturer were adhered (absence of active middle ear infections; ability to get benefit from amplification; ear anatomy allows FMT positioning; stable BC thresholds ≤45 dB HL at 0.5 kHz, ≤50 dB HL at 1 kHz, ≤55 dB at 1.5 kHz, and ≤65 at 2, 3, and 4 kHz). Patients suffering from retro-cochlear, or central auditory disorders as well as patients suffering from conditions that would interfere with the ability to adequately perform the psychoacoustic tests were excluded from the study. If postoperative BC thresholds deteriorated by more than 10 dB compared to preoperative BC thresholds, the patients were excluded from further analysis as well. Informed written consent was obtained from all participants for being included in the study. The study took place at the University Hospital Halle (Saale), Germany and the Friedrichshain Clinic, Vivantes Hearing Center, Berlin, Germany. The protocol was approved by the ethical committee of the Medical Faculty of the Martin Luther University Halle-Wittenberg (approval number 2018-34) and the study was conducted in accordance with the Declaration of Helsinki.

Patients were grouped according to biomechanically comparable FMT coupling modalities to the OW (*OW placement* subgroup), to the incus *(Incus placement* subgroup), and to the RW (*RW placement* subgroup). The OW placement subgroup included all patients with coupling to the stapes suprastructure via a CliP-Coupler or via a Symphonix-Coupler (modified and off-label use for coupling to the stapes head and anterior crus), or to the stapes footplate via an OW-Coupler. All patients with SP-, LP-, or Symphonix-Couplers with standard use (incus vibroplasty) were included in the Incus placement subgroup. The RW placement subgroup included all patients with RWS- (round window soft), or RW-Couplers as well as direct coupling to the RW without a specific coupler (RW vibroplasty).

### Pure-Tone Audiometry

Before surgery, the patients' AC and BC thresholds (preoperative AC and BC) were measured as behavioral pure-tone thresholds at frequencies of 0.5, 1, 2, 3, 4, and 6 kHz with clinical routine audiometers and transducers (circumaural headphones and BC transducers) in a soundproof booth. Approximately 6 weeks after surgery, BC pure tone thresholds and the VIB thresholds were measured as behavioral thresholds using Symfit fitting software (MED-EL, Innsbruck, Austria) within Connexx software (Sivantos GmbH under Trademark License of Siemens AG, Erlangen, Germany) and a Samba Lo audio processor (MED-EL, Innsbruck, Austria). Pure-tone thresholds at 0.5, 1, 2, and 4 kHz were averaged (4PTA_AC_, 4PTA_BC_, 4PTA_VIB_,). Coupling efficiency was determined by computing the difference of postoperatively measured VIB threshold minus BC thresholds, averaged over frequencies of 1, 2, and 4 kHz (3PTA_coupling_).

### VSB Evoked ABR Recordings

VSB evoked ABRs were recorded intraoperatively after positioning of the FMT as described in Froehlich et al. (27). Broadband CE-Chirps were generated by an Eclipse EP25 (Interacoustics A/S, Middelfart, Denmark) clinical auditory evoked potential stimulation and recording system. The acoustic stimuli were delivered at a rate of 49.1 Hz and alternating polarity via a sound tube of EAR-3A insert earphones (3 M, St. Paul, MS, USA) attached to the microphone aperture of an AP404 audio processor (MEDL-EL, Innsbruck, Austria). The gain of the audio processor was set according to the patients' preoperative BC thresholds. The output limitation, compression, and special options (noise reduction, speech enhancement features, etc.) were deactivated.

ABRs were recorded in a two-channel set-up. Self-adhesive surface electrodes were placed at the hairline (active), ~1 cm below this electrode (ground), and on the contralateral mastoids (reference) of the patients. The ipsilateral reference electrode was located at the neck to provide adequate distance to the surgical field. All impedances were kept below 5 kΩ. The EEG signal was sampled at 30 kHz with an A/D resolution of 16 bits and bandpass-filtered between 33 Hz and 1.5 kHz. Epochs with an RMS amplitude below the artifact level of 40 μV were averaged to at least 1,000 stimuli or less, if the residual noise was below 40 nV.

All stimulation levels in the described experimental set-up will be provided in “dB nHL” according to the intrinsic calibration of the Eclipse system. The stimulus intensity level (stimulation level) was increased in steps of 10 dB, or 5 dB close to the ABR threshold and wave V was identified. The lowest level that reproducibly evoked an ABR response was defined as VSB evoked ABR threshold (L_ABR_).

### Data Analysis

Descriptive statistics were used to report pre- and postoperative 4PTA_AC_ and 4PTA_BC_ thresholds, as well as intraoperative L_ABR_ and latency data for the different subgroups. Quantitative data were presented as mean and standard deviation (SD).

Individual intensity-latency functions were computed and depicted for each patient in the different subgroups as uncorrected, 3PTA_coupling_-corrected, and L_ABR_-corrected intensity-latency functions. 3PTA_coupling_-corrected intensity-latency functions were computed by subtracting the individual 3PTA coupling efficiency from the original stimulation levels, i.e., shifting the intensity-latency function toward the perceived loudness. L_ABR_-corrected intensity-latency functions were computed by subtracting the individual L_ABR_ (thresholds) from the original stimulation levels. Those intensity-latency functions were aligned to 10 dB nHL, i.e., the lowest stimulation level for which normative intensity-latency functions were available.

Using a Python software script, individual L_ABR_-corrected intensity-latency functions were fitted to the function Latency (ms) = a∧(Stimulation level–b)+c to compute interpolated latencies for all stimulation levels.

Interpolated wave-V latencies at L_ABR_ threshold level were compared between the different coupling modality subgroups by a one-way analysis of variance (ANOVA). Group averages of interpolated intensity-latency functions were computed for the different subgroups. According to the slope of normal latency-intensity functions at large stimulation levels, the lowest stimulation level at which the mean wave-V latency dropped below 0.10 ms/dB was defined as saturation level.

IBM SPSS-Software version 25 (IBM, Ehningen, Germany) was used for all statistical analyses. Alpha was set to 0.05.

## Results

Twenty-four patients (10 female, 14 male) aged between 33 and 81 years (mean: 58 years) were initially included in the study. Two patients were excluded from further analysis due to reduced 4PTA_BC_ by at least 10 dB compared to the preoperatively measured 4PTA_BC_. Table 1 shows the demographic data including FMT-coupling modality (*n* = 13 patients in OW placement subgroup, *n* = 4 patients in Incus placement subgroup, and *n* = 5 patients in RW placement subgroup) and audiological results. For all patients included in the final analysis (*n* = 22), VSB evoked ABRs could be measured intraoperatively with mean VSB evoked ABR thresholds (L_ABR_) of 6.2 (SD: 5.6) dB nHL (OW placement), 11.0 (SD: 2.2) dB nHL (Incus placement), and 12.04 (SD: 4.0) dB nHL (RW placement).

**Table 1:** Patients' demographics and audiological results.

							**Preoperative**		**Intraoperative**	**Postoperative**	
**ID**	**Age [years]**	**Sex**	**Side**	**Vibroplasty**	**Coupler**	**Reason for Implantation/Pathology**	**4PTA_**AC**_**	**4PTA_**BC**_**	**L_**ABR**_**	**4PTA_**BC**_**	**4PTA_**VIB**_**
							**[dB HL]**	**[dB HL]**	**[dB nHL]**	**[dB HL]**	**[dB]**
“OW placement” group (*n* = 13)											
1	56	Female	R	PORP vibroplasty	CliP-Coupler	Multiple canaloplasties, stenosis of external auditory canal, recurrent OE	51	25	10	19	31
2	51	Male	L	OW vibroplasty	no coupler	Multiple ME surgeries, ME fibrosis	56	28	20	28	44
3	53	Male	L	PORP vibroplasty	CliP-Coupler	Multiple ME surgeries, initial stapes vibroplasty, revision with RW vibroplasty, FMT dislocation, revision	60	44	0	48	45
4	60	Male	L	Stapes vibroplasty	Symphonix on stapes	Previous ME surgery, ME fibrosis, recurrent OE and myringitis with HA	76	44	5	41	54
5	72	Male	L	PORP vibroplasty	CliP-Coupler	Multiple ME surgeries, CWD, ME fibrosis	65	25	0	33	45
6	58	Female	R	TORP vibroplasty	OW Coupler	re-implantation after VORP implant protrusion through skin	58	39	5	39	54
7	60	Male	L	TORP vibroplasty	OW Coupler	Recurrent cholesteatoma, multiple FMT repositioning, FMT dislocation, revision	81	44	5	35	49
8	81	Male	L	PORP vibroplasty	CliP-Coupler	Multiple ME surgeries, chronic OM	80	40	10	34	49
9	52	Female	L	PORP vibroplasty	CliP-Coupler	Multiple ME surgeries, ME fibrosis, atelectasis	58	13	10	14	30
10	67	Male	L	TORP vibroplasty	OW Coupler	Multiple ME surgeries, cholesteatoma, ME fibrosis	83	43	5	51	60
11	67	Male	L	TORP vibroplasty	OW Coupler	Stenosis of external auditory canal, chronic OM and OE	55	34	0	31	38
12	55	Male	R	PORP vibroplasty	CliP-Coupler	Microtia	90	38	0	34	59
13	50	Male	R	PORP vibroplasty	CliP-Coupler	Multiple middle ear surgeries, status post BAHA, granulating otitis media, atalectasis and ME fibrosis	95	53	10	54	77
Mean (SD)	60 (9)						70 (14)	36 (10)	6.2 (5.6)	35 (11)	49 (12)
“Incus placement” group (*n* = 4)											
14	59	Male	L	SP Incus vibroplasty	Incus-SP-Coupler	Multiple canaloplasties, stenosis of external auditory canal	54	41	10	39	49
15	59	Female	R	LP Incus vibroplasty	Incus-LP-Coupler	SNHL, unable to use HA due to hyperhidrosis	46	44	10	39	64
16	68	Female	L	LP Incus vibroplasty	Incus-LP-Coupler	Multiple ME surgeries, PIMF, recurrent otiris externa when using HA	65	34	15	34	45
17	63	Female	L	SP Incus vibroplasty	Incus-SP-Coupler	Chronic otitis externa when using HA	51	34	10	38	38
Mean (SD)	60 (4)						54 (7)	38 (4)	11 (2.2)	37 (2)	49 (9)
“RW placement” group (*n* = 5)											
18	50	Male	L	RW vibroplasty	RW Soft Coupler	Multiple ME surgeries, CWD, ME fibrosis	59	24	15	21	39
19	51	Female	L	RW vibroplasty	no coupler (cartilage)	Multiple ME surgeries, recurrent OE, initial stapes vibroplasty, revision	99	44	5	39	60
20	33	Female	R	RW vibroplasty	RW coupler	VORP implant migration with FMT dislocation, revision	76	38	10	33	31
21	67	Male	L	RW vibroplasty	no coupler	Multiple ME surgeries, ME fibrosis, arrosion of stapes footplate and PL fistula OW	88	48	15	48	65
22	60	Female	L	RW vibroplasty	RW coupler	Multiple ME surgeries, lateral petrosectomy, PL fistula OW	108	51	15	49	57
Mean (SD)	52 (11)						86 (17)	41 (10)	12 (4)	38 (10)	50 (13)

*4PTA, Pure-tone average at 0.5, 1, 2, 4 kHz; AC, Air-conduction; BC, Bone-conduction; CWD, Canal wall down; dB HL, Decibel hearing level; dB nHL, Decibel normalized hearing level; FMT, floating mass transducer; HA, hearing aid; L, Left; L_ABR_, threshold of Vibrant Soundbridge evoked auditory brainstem response; LP, long process; ME, middle ear; OE, otitis externa; OM, otitis media; OW, oval window; PIMF, postinflammatory meatal fibrosis; PL, perilymph; PORP, partial ossicular replacement prosthesis; R, Right; RW, round window; SD, Standard deviation; SNHL, sensorineuroal hearing loss; SP, short process; TORP, total ossicular replacement prosthesis; VORP, vibrating ossicular replacement prosthesis*.

**Figure 1:**
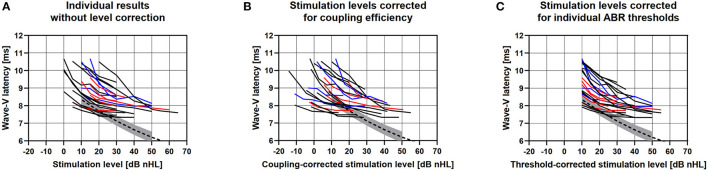
**(A)** Individual VSB evoked ABR wave-V latencies for all applied stimulation levels of all included patients with *OW placement* (black), *Incus placement* (blue), and *RW placement* (red). The dashed curves show the mean wave-V reference data of an acoustic click stimulation for the ABR system used in the study. Gray areas show the standard deviations. **(B)** Individual wave-V latencies after level correction for coupling efficiency, i.e., reduction by 3PTA_VIB_ minus 3PTA_BC_. **(C)** Individual wave-V latencies after correction for the individual ABR threshold, aligned to a stimulation level of 10 dB nHL.

Wave-V latencies could be discerned in all patients. Figure 1A shows the individual intensity-latency functions. Close to the individual L_ABR_, wave-V latency decreased with increasing stimulation level with a slope comparable to that of reference latency data for acoustic stimulation (37–39). At stimulation levels of 30 dB and more above L_ABR_, the slopes converged into a steady-state latency of 7–8 ms. Individual wave-V intensity-latency functions as level-corrected by the individual 3PTA_coupling_ are shown in Figure 1B. Figure 1C shows the L_ABR_-corrected wave-V intensity-latency functions by aligning the lowest stimulation level with a clear wave-V response to 10 dB nHL. At individual threshold level L_ABR_, wave-V latencies showed a large interindividual variance. For increasing stimulus levels, the variance decreased with intensity-latency functions converging toward the saturation latency.

**Figure 2:**
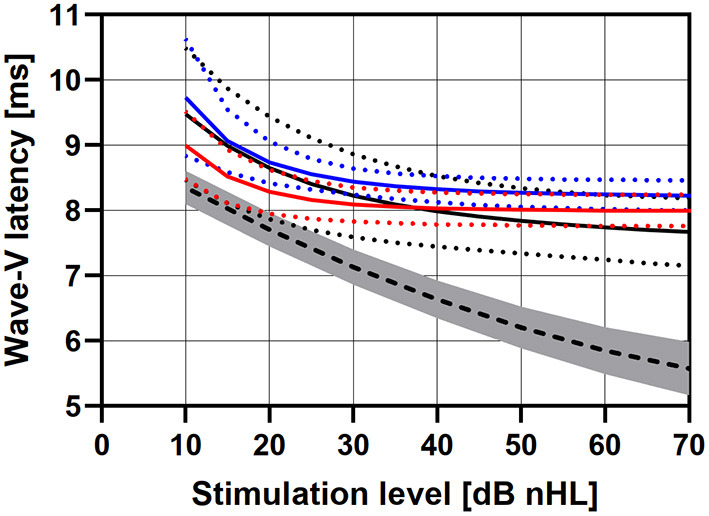
VSB evoked ABR wave-V latencies as group averages for subgroups of *OW placement* (black), *Incus placement* (blue), and *RW placement* (red). Reference data for an acoustic click stimulation are shown as dashed line. The dotted lines, errors bars, and the gray area depict standard deviations.

The mean intensity-latency functions for all patients and subgroups of OW-, Incus-, and RW placement, were computed from the interpolated L_ABR_-corrected intensity-latency functions and depicted in Figure 2. Over all patients and subgroups, the wave-V latencies were largest at 10 dB nHL (9.4 ms; SD: 0.93) and decreased with increasing stimulation levels to 8.06 ms (SD: 0.46) at 40 dB nHL and 7.84 ms (SD: 0.49) at 70 dB nHL. At threshold level L_ABR_, the wave-V latency was longest in the Incus placement subgroup (9.73 ms, SD: 1.04), followed by the OW placement subgroup (9.47 ms, SD: 1.05), and the shortest latency was observed in the RW placement subgroup (8.99 ms, SD: 0.68). The reference for acoustic stimulation, i.e., wave-V latency for acoustic click stimulation, was 8.35 ms (SD: 0.55) at 10 dB nHL. The effect of coupling modality subgroup on wave-V latency at threshold level L_ABR_ was not statistically significant [*F*_(2,19)_ = 0.706, *p* = 0.506]. In the RW placement subgroup, mean wave-V latencies were lower as compared with the Incus placement subgroup at all stimulation levels. Latency saturation was reached at a stimulation level of 50 dB nHL for the OW placement subgroup, 35 dB nHL for the Incus placement subgroup, and 30 dB nHL for the RW placement subgroup.

## Discussion

The results from our study showed no correlation of VSB evoked ABR wave-V intensity-latency functions with respect to coupling efficiency (see Figure 1B) and rather large variance of the individual wave-V latencies at threshold level L_ABR_. However, it was observed that intensity-latency function slopes converged into a steady-state latency of 7–8 ms at stimulation levels between of 30 dB and more above L_ABR_. This is in contrast to acoustic stimulation where the latencies continue to decrease with increasing stimulation levels. Saturation in VSB evoked ABR wave-V latencies most likely occurred to due limited dynamic range of the audio processor used for signal transmission. It was already reported in the study investigating the relation between L_ABR_ and coupling efficiency that the audio processor output saturated between 35 and 40 dB nHL stimulation level depending on gain settings [see Figure 1 in (27)]. Stimulation levels at threshold level L_ABR_ were perceived by the patient as very soft sounds. Accordingly, the perceived loudness by the patient did not increase with saturation of output by the audio processor, resulting in saturation of ABR latencies. Thus, the analysis of VSB evoked ABR wave-V intensity-latency functions enables the objective prediction of dynamic range for the individual patient treated with the AMEI.

It was observed that the interpolated L_ABR_-corrected intensity-latency functions showed differences between wave-V latencies at L_ABR_ with respect to coupling modality although not statistically significant. However, the difference between latencies at high stimulation levels above saturation level was unexpected and most likely caused mathematically by the applied approximation functions. A saturation at equal latencies was expected based on technical limitations (see above). Shorter latencies and lower levels for saturation as found for the RW placement group in comparison to the OW- and Incus placement group indicated differences in signal transmission between the different coupling modalities. That is, a lower dynamic range was observed for patients with reverse stimulation for RW placement especially in comparison to OW placement. This is clinically significant as it suggests that there is a difference between activation of the auditory system by direct stimulation in the natural normal direction via the oval window—similar to acoustic stimulation—and reverse stimulation via the RW. The method could therefore be used to investigate the clinical implications of different coupling modalities. The total offset between VSB evoked wave-V latencies and normative data for acoustic stimulation was most likely caused by a signal processing delay in the audio processor. Reference data for acoustic click stimulation were used here due to technical measurements which showed that the CE-chirp used for stimulation emerged as a click-like stimulus after transmission through the audio processor and VSB (24, 27).

The finding of reduced dynamic range in RW coupling modalities is of special clinical interest and complements findings in other studies showing that RW placement is surgically and technically less favorable compared to OW placement or Incus placement. With respect to coupling efficiency, OW and Incus placement showed better efficiency (smaller differences between VIB and BC thresholds) compared to RW placement in a study by Müller et al. (18). If the loss of energy transmission to the inner ear cannot be compensated technically by adjusting gain settings, insufficient coupling can result in the need for revision surgeries. Data from the literature for revision surgeries in RW vibroplasty vary between 9.5% (43) and 29.0–71.0%, depending on the use of couplers (44). In comparison to other coupling modalities, RW coupling showed the highest complication rates (52.6%) in a study by Brkic et al. (45). Postoperative BC deterioration is one of the problems reported for RW vibroplasty. Data in the literature vary between 3.0% (46) and 20.0% in patients with RW vibroplasty (compared to 11.1% of patients with incus vibroplasty) (47).

Comparison between acoustic stimulation and electromechanical stimulation by an FMT has only been performed in experimental studies using Laser-Doppler vibrometry so far. These studies provided objective evidence that acoustic stimulation is mechanically different from electromechanical stimulation by the VSB (9, 10). However, normative data on the characteristics of VSB evoked ABRs as a new objective method were not available in the literature so far. Thus, comparison of our data to data in the literature is limited because only very few studies reported AMEI evoked wave-V latencies. Verhaert et al. (42) observed interindividual wave-V latency differences in their study. This is in line with our findings although comparison is limited due to the difference between AMEIs investigated in the studies and the experimental stimulation set-ups applied for signal transmission. Generally, variability in latency is also affected by hearing loss, age, and sex among other factors (48, 49).

In summary, the results showed that the measurement of VSB evoked ABR wave-V intensity-latency functions allows for the assessment of the patient's individual dynamic range as well as comparison between coupling modalities. In this aspect, it is similar to measuring transfer functions for different coupling modalities but with respect to the actual coupling situation in the individual patient. Thus, postoperative recordings of VSB evoked intensity-latency functions could presumably be used to qualitatively assess coupling efficiency within one patient. With the transmission characteristics, i.e., the coupling efficiency, changing over time, a change of intensity-latency functions would be expected. Thus, VSB evoked ABR wave-V intensity-latency functions may be used for postoperative quality control in follow-up visits where the patients serve as their own control.

One of the major limiting factors of the current study is the limited number of data points for wave V-latencies, i.e., the limited range of stimulation intensities for which VSB evoked ABRs were recorded. More data points for higher stimulation levels above threshold level have to be recorded in future investigations to acquire data for a larger dynamic range. The different number of patients in each subgroup was another factor limiting the interpretation of the data. Both, the limited number of data points and unequal size of subgroups, account for the explorative, hypotheses generating nature of the current study. Another challenging factor was the multidimensionality of the analysis, as intensity-latency functions could depend on various different factors such as the patients' hearing loss, i.e., dynamic range provided by the audio processor by its gain settings, the coupling efficiency, the coupling modality, and the frequency specific output of the VSB itself. For the analysis, we concentrated on high frequencies between 1 and 4 kHz (3PTA) where contribution to signal transmission with the VSB is largest. The current study only concentrates on CE-Chirp stimulation which results in click-like stimulation with the VSB. The use of a VSB specific chirp as suggested by Cebulla et al. (24) may give different results. Besides these limitations, the current study only focuses on the analysis of intensity-latency functions but does not relate the findings to postoperative speech reception. This should be part of future research especially with respect to the prediction of the patient's dynamic range, which—besides coupling efficiency—significantly influences speech reception (50, 51). The findings should also be complemented by measuring the patient's individual dynamic range behaviorally by loudness scaling methods.

## Conclusion

In conclusion, the data show that VSB evoked wave-V intensity-latency function slopes were similar to acoustic stimulation at high stimulation levels. Absolute latencies were longer compared to acoustic stimulation, most likely caused by a signal processing delay in the VSB audio processor. Saturation of latencies occurred for higher stimulation levels due to saturation of audio processor gain. Thus, the analysis of VSB evoked intensity-latency functions can be useful for the objective assessment of a patient's individual dynamic range with the AMEI. Wave-V latencies were found to be longer for Incus and OW placement of the FMT compared to RW placement, i.e., reverse stimulation, showing differences in activation of the auditory system for different coupling modalities.

In summary, the analysis of VSB evoked ABR wave-V intensity-latency functions likely enables the objective quantification of coupling efficiency by the response threshold L_ABR_, providing a patient with the maximum possible individual dynamic range with the AMEI. In addition, it may become a useful tool for the objective assessment of the patient's individual dynamic range, the comparison of different coupling modalities and continuous postoperative quality control.

## Data Availability Statement

The datasets generated for this study are available on request to the corresponding author.

## Ethics Statement

The studies involving human participants were reviewed and approved by Ethical Committee of the Medical Faculty of the Martin Luther University Halle-Wittenberg. The patients/participants provided their written informed consent to participate in this study.

## Author Contributions

LF: conceptualization, methodology, formal analysis, investigation, and writing—original draft. TR: conceptualization, methodology, formal analysis, investigation, writing—original draft, and review and editing. PM-S and SP: surgeries. AM, MK, PM-S, SP, OD, and TO: conceptualization, methodology, investigation, and writing—review and editing. All authors contributed to the article and approved the submitted version.

## Conflict of Interest

LF, TR, SP, AM, OD, and TO received travel expenses from MED-EL (Innsbruck, Austria). SP received speaker honorarium on bone conduction hearing devices (including a different product from MED-EL, Innsbruck) and conducted contract research for MED-EL, Innsbruck, on cochlear implants. The remaining authors declare that the research was conducted in the absence of any commercial or financial relationships that could be construed as a potential conflict of interest.

## Publisher's Note

All claims expressed in this article are solely those of the authors and do not necessarily represent those of their affiliated organizations, or those of the publisher, the editors and the reviewers. Any product that may be evaluated in this article, or claim that may be made by its manufacturer, is not guaranteed or endorsed by the publisher.

## Abbreviations

AMEI: active middle ear implant
VSB: Vibrant Soundbridge
FMT: floating mass transducer
LP: long process (of the incus)
SP: short process (of the incus)
OW: oval window
RW: round window
VIB: vibrogram (thresholds)
BC: bone conduction (thresholds)
ASSR: auditory steady state response
CAP: compound action potential
ABR: auditory brainstem response
3PTA_coupling_: pure tone average of coupling efficiency at 1, 2, and 4 kHz
AC: air conduction
4PTA_AC_: pure tone average of air conduction thresholds at 0.5, 1, 2, and 4 kHz
4PTA_BC_: pure tone average of bone conduction thresholds at 0.5, 1, 2, and 4 kHz
4PTA_VIB_: pure tone average of vibrogram conduction thresholds at 0.5, 1, 2, and 4 kHz
L_ABR_: VSB evoked ABR thresholds
SD: standard deviation.
